# The inhibitory effects of ginsenosides on periodontitis pathogenic bacteria

**DOI:** 10.3389/fmicb.2025.1573969

**Published:** 2025-03-26

**Authors:** Qiuyang Lei, Jianrong Chen, Ye Yuan, Chenxing Hu, Zhiying Lin, Shuang Yang, Kevin H. Mayo, Yifa Zhou, Lin Sun, Wenzhi Song

**Affiliations:** ^1^Department of Stomatology, China-Japan Union Hospital, Jilin University, Changchun, Jilin, China; ^2^Engineering Research Center of Glycoconjugates, Ministry of Education, Jilin Provincial Key Laboratory of Chemistry and Biology of Changbai Mountain Natural Drugs, Northeast Normal University, Changchun, China; ^3^Department of Biochemistry, Molecular Biology and Biophysics, University of Minnesota, Minneapolis, MN, United States

**Keywords:** ginsenosides, periodontitis, *Porphyromonas gingivalis*, *Fusobacterium nucleatum*, biofilm

## Abstract

Periodontitis is mainly caused by bacterial destruction of periodontal tissue in dental plaque. Commonly used antibiotic treatment has some shortcomings, such as incomplete sterilization, drug resistance, and local flora imbalance. Because of this, there is a need to identify safe and non-drug resistant health products with high antibacterial activity. Ginsenosides, the primary active component in ginseng, have been shown to be antibacterial. In this study, we investigated the inhibitory effects of ginsenosides on *Porphyromonas gingivalis* and *Fusobacterium nucleatum*, along with their structure–activity relationships and mechanisms of action. Our results show that total ginsenosides elicit a significant inhibitory effect on the growth of periodontal pathogens, with antibacterial effects from PPD-type saponins being greater than those from PPT-type saponins. Among different monomer saponins, Rd had the best antibacterial effect and inhibited the growth of periodontal pathogens at 250 μM. Mechanistic analyses indicated that total ginsenosides mainly function at inhibiting biofilm formation by reducing cell surface hydrophobicity and extracellular polysaccharide content. Our study provides the basis for development of new, plant-based treatment drugs against periodontal disease.

## 1 Introduction

Periodontitis is considered by the World Health Organization (WHO) as a major dental disease. Periodontitis is an inflammation-mediated microbial-host interaction, which eventually leads to periodontal attachment loss. Currently, the most widely recognized periodontal pathogens include *Porphyromonas gingivalis*, *Fusobacterium nucleatum*, *Tannerella forsythia*, *Actinobacillus actinomycetemcomitans*, and *Prevotella intermedia*, among which *P. gingivalis* is the primary etiological agent of chronic periodontitis in adults (Fine et al., 2007). These periodontal pathogenic bacteria and their products in the biofilm are believed to be the initiating factors in periodontal disease. Thus, controlling bacterial infections and eliminating pathogenic factors are key to the prevention and treatment of chronic periodontitis.

Currently, the main clinical treatment includes mechanical debridement, systemic drug therapy, periodontal surgery, among others. Drug treatment is divided into systemic treatment and local drug treatment. Systemic drug therapy mainly includes antibiotics, such as nitroimidazole, tetracycline, and penicillin, while local drug therapy contains minocycline hydrochloride ointment (Cope et al., 2018; Yan et al., 2020). These antibiotics can effectively eliminate bacteria at the bottom of deep periodontal pockets and the root bifurcation area. However, they are prone to inducing side effects, such as intestinal and systemic allergic reactions, but they also can promote drug resistance and destruction of microbial balance (Gomes et al., 2020).

Development of new non-toxic products that avoid drug resistance and are effective at inhibiting periodontal pathogen proliferation and plaque biofilm formation, has become crucial to alleviating periodontal disease. In this regard, plant-derived natural products have recently attracted considerable attention. Natural products such as curcumin (Li et al., 2021), naringin (Chang et al., 2017), baicalin (Sun et al., 2016), can inhibit bacterial growth and reproduction via multiple mechanisms, making it an effective approach to improving periodontal health.

Ginseng is widely used in herbal remedies. The primary pharmacologically active component in ginseng are ginsenosides. Based on differences of sapogenins, ginsenosides are divided into protopanaxadiol, protopanaxatriol and oleanolic acid saponins. Among them, protopanaxadiol and protopanaxatriol belong to dammarane saponins, accounting for the vast majority of ginsenosides (Hou et al., 2021). Protopanaxadiol and protopanaxatriol are tetracyclic triterpenoids that can be decorated by various sugars. Protopanaxadiol-type saponins are PPD decorated with glycosyl groups at their C-3 and C-20 positions, including Rb1, Rb2, Rb3, Rc, Rd, Rg3 (Hou et al., 2021). Protopanaxatriol-type saponins are PPT decorated with glycosyl groups at the C-6 and C-20 positions, including Re, Rf, Rg1, Rg2, Rh1, Rh3. The biological activity of ginsenosides is closely related to its structure. The difference in the type of glycosyl attached to the aglycone skeleton, the substitution site, and the glycosidic bond configuration of the sugar chain length group may be the reason for the difference in biological activity between different components of ginsenosides (Yousof Ali et al., 2021).

Previous studies have found that ginsenosides Rg5 (Xue et al., 2017), Rd (Zhou et al., 2022) and Rg6 (Lee et al., 2024) have significant inhibitory effects on periodontal pathogens, proving that ginsenosides are a potential natural drug for the treatment of periodontitis. However, the relationship between the structure and antibacterial activity of ginsenosides is not yet clear, and further research is needed. In this study, we examined the inhibitory effects from total ginsenosides on the growth of *P. gingivalis* and *F. nucleatum*, along with antibacterial effects from ginsenoside-isolated protopanaxadiol (PPD)-type and protopanaxatriol (PPT)-type saponins.

To assess structure–activity relationships, monomer saponins of the PPD-type (Rb1, Rb3, Rd, Rg3) and PPT-type (Re, Rg1, Rg2, Rh1) were investigated, and their inhibitory effects on the growth of *P. gingivalis* and *F. nucleatum* were compared. Furthermore, the antibacterial mechanisms of these ginsenosides were determined. Our results show that ginsenosides have significant inhibitory effects on periodontitis pathogenic bacteria, thus providing a new strategy to potentially prevent and treat periodontal disease.

## 2 Materials and methods

### 2.1 Materials

Ginseng leaf-stem saponins were purchased from Ningbo Jinainong Biotechnology Co., Ltd. (Ningbo, China). Ginsenoside standards (Rd, Rb1, Rb3, Rg3, Re, Rg1, Rg2 and Rh1) with >98% purity were purchased from CHENGDU MUST BIO-TECHNOLOGY CO., LTD (Chengdu, China). *P. gingivalis* BNCC 236547 and *F. nucleatum* 280,188 were purchased from Suzhou BeNa Culture Collection (BNCC) Biotechnology CO., LTD (Suzhou, China). Other chemicals are of analytical or chromatographic grade.

### 2.2 Preparation of ginsenosides

The total ginsenosides from ginseng stems and leaves were separated into two components (PPD-type and PPT-type ginsenosides) by membrane filtration. The aqueous solution of total ginsenosides was divided into retention and permeation solutions by using hollow fiber ultrafiltration (GE Healthcare UFP-3-C-6A, USA) with a molecular weight cut-off of 3 kD.

The PPD-type ginsenosides (Rb1, Rb3, Rd) and PPT-type ginsenosides (Re, Rg1) were isolated through optimized silica gel column chromatography. This protocol was adapted from our laboratory’s established methodology (Wang et al., 2018) with modifications to the mobile phase system: n-butanol/ethyl acetate/water (4:4:1, v/v/v) for Rb1, Rb3 and Rd, while methanol/ethanol/ethyl acetate mixture (7:3:50, v/v/v) for Re and Rg1. Additional ginsenosides (Rg3, Rh1, Rg2) were produced via our laboratory’s well-characterized enzymatic biotransformation (Hu et al., 2019; Zhao et al., 2012; Yuan et al., 2015; Hu et al., 2024).

### 2.3 Identification of ginsenoside components

Ginsenoside components as isolated above, were identified and purified by using high performance liquid chromatography (HPLC). Detection conditions used are as previously reported (Hu et al., 2024). The sample injection volume was 10 μL. The liquid phase consisted of a Waters 2,695 separation unit and a 2,998 secondary tube array detector. A C18 chromatographic column (4.6 × 250 mm, 5 μm) was used to separated sample components. The mobile phase was acetonitrile (A) and ultrapure water (B). The elution program was: 0–5 min 70% A; 6.3–15 min 65% A; 15–25 min 60% A; 25–50 min 20% A; 50–60 min 0% A; 80–100 min 70% A. The column temperature was set at 30°C, and the elution flow rate was 0.4 mL/min. The absorption wavelength of 203 nm was measured, and the type of ginsenoside was determined by retention time compared to that of standard saponins.

### 2.4 Bacterial strain and growth conditions

On an ultra-clean bench, 0.5 mL of sterile water was taken into disinfected ampoule bottles containing *P. gingivalis* and *F. nucleatum*, and 200 μL bacterial solution was uniformly coated onto the Colombian blood plates followed by slow blowing and mixing. Blood plates inoculated with bacteria were placed in an anaerobic culture jar (Gene Science anaero station AG300, USA) at 37°C under anaerobic conditions (10% CO_2_, 10% H_2_, and 80% N_2_). *P. gingivalis* was cultured for 10 days, and *F. nucleatum* was cultured for 4 days.

Single colonies were cultured in modified Brain Heart Infusion Broth (BHI medium) composed of 10 g/L peptone, 12.5 g/L dehydrated calf brain extract powder, 5.0 g/L dehydrated bovine heart extract powder, 5.0 g/L NaCl, 2.0 g/L Glucose, 2.5 g/L Na_2_HPO_4_, 1% hemin, and 0.1% Vitamin K_1_ at 37°C for 24 h under anaerobic conditions in an anaerobic culture jar. After culturing, the two strains were identified by Matrix-assisted laser desorption/ionization time-of-flight mass spectrometry (MALDI-TOF-MS) (Bruker autoflex speed MALDI TOF/TOF, Germany).

The concentration of *P. gingivalis* and *F. nucleatum* was adjusted to 1 × 10^8^ CFU/mL using a nephelometer for subsequent experiments.

### 2.5 Determination of minimum inhibitory concentrations

The minimum inhibitory concentration (MIC) and minimum bactericidal concentration (MBC) were determined by using the micro-double dilution method (Wang et al., 2013). BHI liquid medium was used to dilute the total ginsenoside solution. 96-well microplates were inoculated with 100 μL diluted suspension of *F. nucleatum* and *P. gingivalis* in each well, and then 100 μL samples with different concentrations were added. The range of dilutions of total ginseng saponins were 4 to 0.03125 mg/mL in the 96-well microplates. The BHI bacterial suspension culture medium without ginseng total saponin solution was used as the negative control, and the bacterial suspension containing 20 μg/mL ornidazole solution and experimental bacteria was used as the positive control. Plates were incubated in an anaerobic incubator at 37°C for 24 h and then the absorbance at 600 nm was detected by using a microplate reader (Tecan infinite F50, Switzerland). MIC is defined as the minimum concentration of OD_600_ change ≤0.05 compared with the control group. All determinations were performed in triplicate.

For detection of MBC of the total ginsenoside, each well with a concentration greater than or equal to the MIC was selected, and 20 μL of mixed culture solution was coated on a Colombian blood plate. *P. gingivalis* was cultured in an anaerobic incubator at 37°C for 120 h, and *F. nucleatum* was cultured for 72 h. The lowest concentration with fewer than 5 to 6 colonies is defined as the MBC. All determinations were performed in triplicate with three replicates in each group.

### 2.6 Detection of DMSO toxicity

To exclude the effect of DMSO on bacterial growth, toxic effects of different concentrations of DMSO on *P. gingivalis* and *F. nucleatum* were examined. The final concentrations of DMSO were 5, 4, 3, 2, 1, 0.5 and 0%, achieved by adding bacterial suspensions and different volumes of DMSO in 96-well plates. The concentration of the bacterial suspension was diluted to 1 × 10^8^ CFU/mL ahead of time. Plates were then placed in an anaerobic incubator for 24 h, and the absorbance at 600 nm was detected by using a microplate reader. The experiment was repeated three times independently, with three parallels in each group.

### 2.7 Inhibitory effects of PPD- and PPT-type saponins

In order to understand the structure–activity relationship of ginsenosides against periodontal pathogens, MIC values of PPD- and PPT-type saponins against *P. gingivalis* and *F. nucleatum* were determined, and their antibacterial effects were compared. *P. gingivalis* and *F. nucleatum* bacterial suspensions were transferred to 96-well plates with different concentrations of PPD- and PPT-type saponins in each group. The reaction systems are the same as described in the previous section. In order to compare the inhibitory effect of different ginsenoside components on periodontal pathogens at the same concentration, the final concentration of PPD-type monomer saponins and PPT-type monomer saponins was consistent with that of total ginsenosides.

After 24 h of culture under anaerobic conditions, the OD_600_ was detected and used to determine the antibacterial effect. The experiment was repeated three times independently, with three parallels in each group.

### 2.8 Inhibitory effects of different monomer saponins

To explore structure–activity relationships of ginsenosides, PPD monomer saponins Rb1, Rb3, Rd, Rg3 and PPT monomer saponins Re, Rh1, Rg1, Rg2, were used to detect their inhibitory effects on *P. gingivalis* and *F. nucleatum*. The antibacterial activity of all monomer saponins was determined at three concentrations of 500 μM, 250 μM and 50 μM (Zhou et al., 2022). Samples were dissolved in 1.5% DMSO and diluted with BHI medium to 500 μM, 250 μM and 50 μM. *P. gingivalis* and *F. nucleatum* bacterial suspensions were transferred to 96-well plates with different concentrations of monomer saponins in each group. The culture system and conditions were as described in the previous section. Experiments were repeated three times independently, with three parallels in each group.

### 2.9 Crystal violet assay

The biomass of biofilms after treatment of *P. gingivalis* and *F. nucleatum* with different concentrations of total saponins were determined as previously reported with slight modifications (Zhou et al., 2022). The concentration of *P. gingivalis* and *F. nucleatum* was adjusted to 1.0 × 10^6^ CFU/mL. Bacterial stains were treated with total ginseng saponins (1/8×, 1/4×, 1/2×, 1× and 2× MIC) in a 24-well plate with coverslips for 3 d in the dark. The control group was BHI liquid medium without ginsenosides. After culturing, coverslips with adherent bacteria were washed with phosphate buffered saline (PBS) for 3 times, dried at room temperature, and fixed with 200 μL 2.5% glutaraldehyde for 20 min. Subsequently, 200 μL 0.1% crystal violet reagent was added for 20 min, and then the excess crystal violet reagent was washed with PBS. Staining of the bacterial biofilm was observed under a microscope (Novel NSZ608T, China). Experiments were repeated three times independently, with three parallels in each group.

### 2.10 Determination of cell surface hydrophobicity of *Porphyromonas gingivalis* and *Fusobacterium nucleatum*

The cell surface hydrophobicity (CSH) of *P. gingivalis* and *F. nucleatum* treated with different concentrations of total ginsenosides was determined by using the microbial hydrocarbon adhesion (MATH) method (Drozd and Schwartzbrod, 1996). Specifically, stains *P. gingivalis* and *F. nucleatum* were treated with the total ginseng saponins (1/4× and 1/2× MIC) under anaerobic conditions at 37°C for 6 h and 12 h. After that, cultures were centrifuged at 5500 rpm for 10 min to collect the precipitate, that was resuspended in 500 μL phosphate urea magnesium (PUM) buffer. OD values were spectrophotometrically measured at 500 nm (A_0_). Next, measured solutions were transferred back to the centrifuge tube, and 100 μL of N-hexadecane was added to each tube. After oscillation, solutions were allowed to stand for 15 min, during which the solution in the centrifuge tubes separated into two layers. The lower one was the buffer layer, and 100 μL of this layer was transferred to the 96-well plate. OD values were then measured at 500 nm (A_1_). CSH was represented as the hydrophobic index: [(A_0_ − A_1_)/A_0_] × 100%. All sets were done in triplicate.

### 2.11 Determination of extracellular polysaccharides content in biofilms

Extracellular polysaccharides (EPS), a main component in biofilms, promotes formation of a cohesive three-dimensional framework (Pan et al., 2016). The content of EPS in the biofilm was determined by using the phenol-sulfuric acid method. *P. gingivalis* and *F. nucleatum* were treated with different concentrations of total ginseng saponins in a 24-well plate with coverslips for 72 h (Felz et al., 2019). Bacterial concentrations were adjusted to 1 × 10^6^ CFU/mL. The total ginsenoside solution was dissolved in BHI liquid medium according to the half dilution method, so that final concentrations were 1.0, 0.5, 0.25, 0.125, 0.0625, 0.03125 mg/mL. The control group was BHI liquid medium without ginsenosides. Coverslips with adherent bacteria were washed with PBS for 3 times. After drying at room temperature, 80 μL of distilled water, 80 μL 6% phenol and 400 μL 97% concentrated sulfuric acid were added in turn, and the reaction was carried out for 20 min. The absorbance at 490 nm was detected by using a microplate reader. Experiments were repeated three times independently, with three parallels in each group.

### 2.12 Statistical analysis

Experimental data were statistically analyzed using GraphPad Prism 8 software, and multiple comparisons were made between experimental and control groups by using the standard one-way analysis of variance. The difference between experimental and control groups was considered statistically significant when *p* < 0.05.

## 3 Results and discussion

### 3.1 Preparation and identification of ginsenoside components

**Figure 1:**
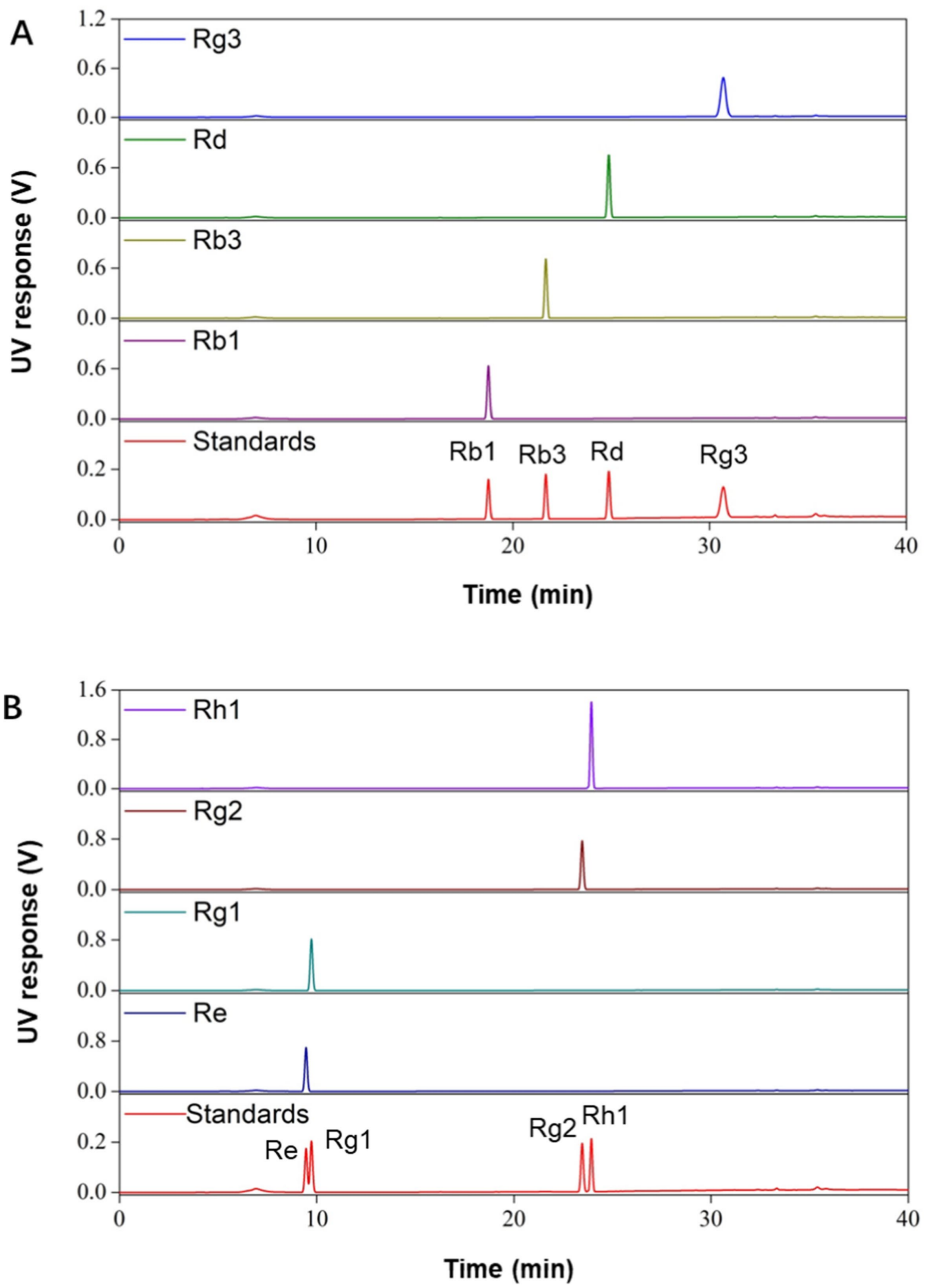
HPLC analysis of different monomer ginsenosides. **(A)** HPLC analysis of PPD-type saponins. **(B)** HPLC analysis of PPT-type saponins.

In this study, we prepared a series of ginsenoside components and monomers from total saponins in ginseng stems and leaves. Total saponins were separated into PPD- and PPT-type rich components by using membrane filtration. The composition and content of monomer saponins were detected by HPLC as shown in Table 1. The retention component was mainly composed of PPD-type saponins (83.3%), and the permeation component was mainly composed of PPT-type saponins (81.9%). Among the PPD-type saponins, the content of Rd was the highest (34.6%), and the content of Rb3 was the lowest (3.3%). PPT-type saponins were mainly composed of Re (43.6%). In addition, monomer ginsenosides Rb1, Rb3, Rd, Rg1, Rg2, Rg3, Re and Rh1 were also prepared, with HPLC detection results shown in Figure 1. The purity of all monomer ginsenosides were > 90%.

**Table 1:** Composition and content of PPD- and PPT-type saponins.

	Retention component	Permeation component
Re	9.5%	43.6%
Rg1	3.4%	19.0%
Rb1	10.6%	1.9%
Rc	8.2%	-
Rb2	9.8%	7.3%
Rg2	-	9.1%
Rh1	-	3.0%
Rb3	3.3%	-
Rd	34.6%	5.8%
F1	3.7%	7.2%
C-O	4.5%	1.1%
C-MC1	7.2%	2.0%
C-MX1	5.1%	-
Proportion of PPD-type saponins	83.3%	18.1%
Proportion of PPT-type saponins	16.6%	81.9%

### 3.2 Identification of *Porphyromonas gingivalis* and *Fusobacterium nucleatum*

**Figure 2:**
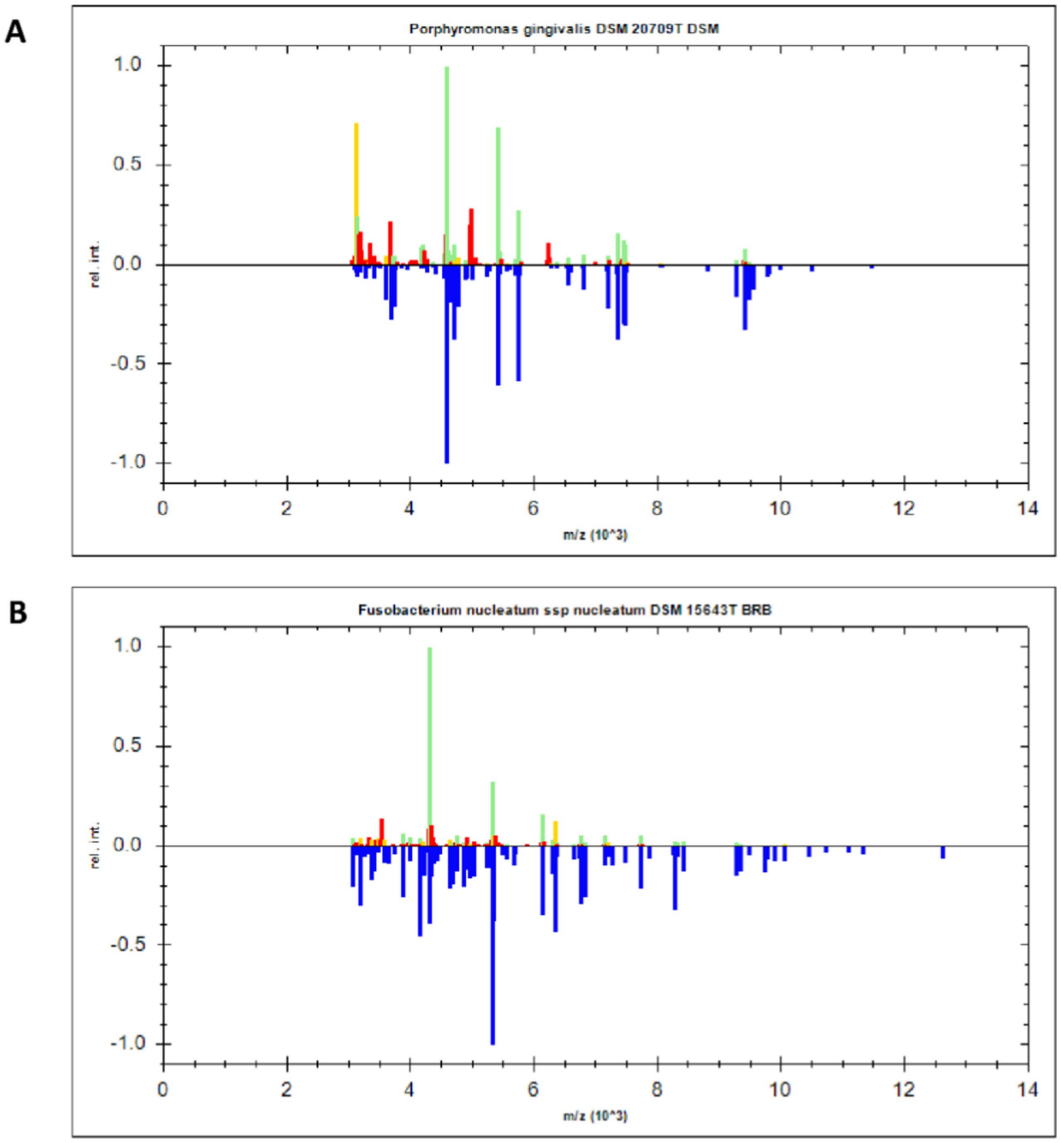
MALDI-TOF-MS identification of *P. gingivalis* and *F. nucleatum*. **(A)** The specific protein peak of *P. gingivalis*. **(B)** The specific protein peak of *F. nucleatum*.

MALDI-TOF-MS can determine the bacterial species by analyzing peaks of specific proteins from different bacteria (Zhao et al., 2024). Here, MALDI-TOF-MS was used to identify two periodontal pathogenic bacteria strains after resuscitation, with mass-spectrograms being compared with strains in the database. As shown in Figure 2, two strains were identified as *P. gingivalis* DSM 20709 T DSM and *F. nucleatum ssp nucleatum* DSM 15643 T BRB. These strains were used in our subsequent experiments.

### 3.3 Antibacterial effects of total ginsenosides on *Porphyromonas gingivalis* and *Fusobacterium nucleatum*

**Figure 3:**
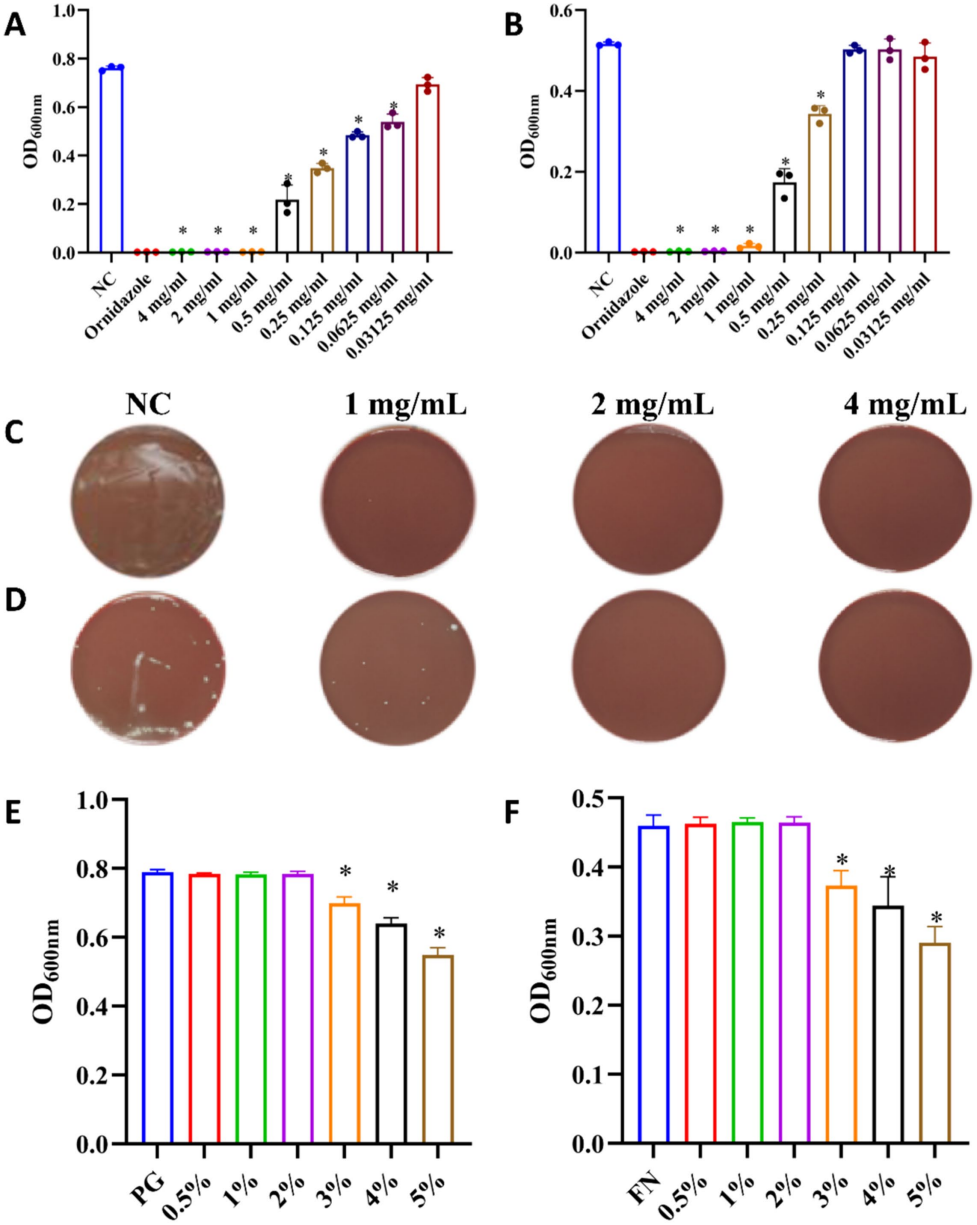
Antibacterial effects of total ginsenosides. **(A)** MIC of total ginsenosides toward *P. gingivalis*. **(B)** MIC of total ginsenosides toward *F. nucleatum*. **(C)** MBC of total ginsenosides toward *P. gingivalis*. **(D)** MBC of total ginsenosides toward *F. nucleatum*. **(E)** Toxic effects of DMSO on *P. gingivalis.* **(F)** Toxic effects of DMSO on *F. nucleatum.* *p < 0.05 versus the normal control.

The antibacterial effect of total ginsenosides was detected by measuring MIC and MBC values. As shown in Figures 3A,C, the MIC and MBC values of total ginsenosides toward *P. gingivalis* were both 1 mg/mL, whereas MIC and MBC values of total ginsenosides against *F. nucleatum* were 1 mg/mL (Figure 3B) and 2 mg/mL (Figure 3D), respectively. In Figures 3A,B, 0.25 mg/mL and 0.5 mg/mL total ginsenosides significantly inhibited the growth of *P. gingivalis* and *F. nucleatum*. Total ginsenosides at 1–4 mg/mL completely inhibited the growth of both bacteria, and the number of bacterial colonies was significantly reduced. In order to exclude interference from solvents, the toxicity of DMSO alone to *P. gingivalis* and *F. nucleatum* was detected. As shown in Figures 3E,F, addition of 0.5 to 2% DMSO had no effect on bacterial growth. When the DMSO concentration reached 3%, the growth of *P. gingivalis* and *F. nucleatum* was partially inhibited. Because of this, we held the DMSO concentration less than 1.5% in all experiments. Overall, total ginsenosides exhibited a significant inhibitory effect on the growth of *P. gingivalis* and *F. nucleatum.*

### 3.4 Inhibitory effects of PPD- and PPT-type saponins

**Figure 4:**
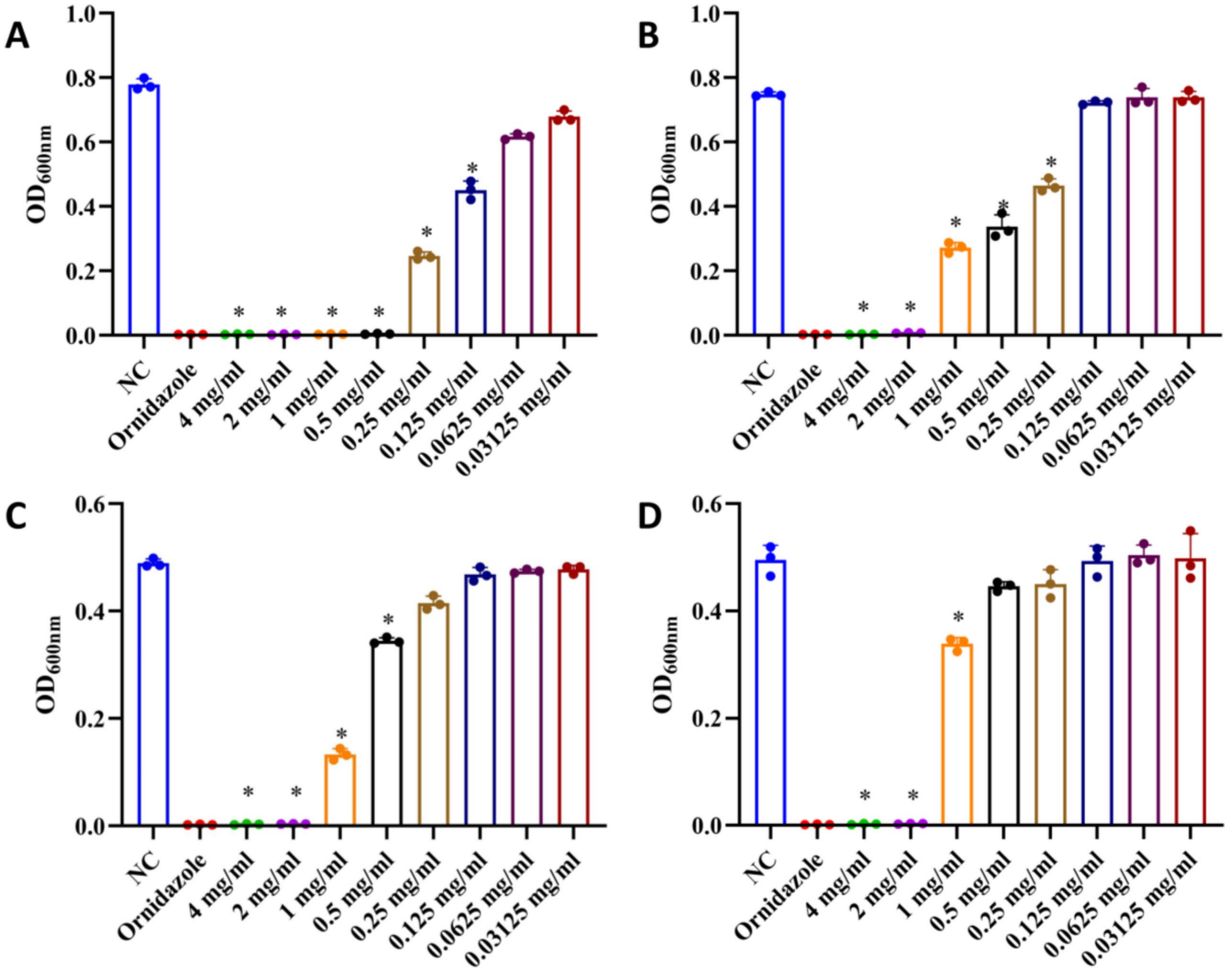
Effects of PPD- and PPT-type saponins on the growth of *P. gingivalis* and *F. nucleatum*. **(A)** Inhibitory effect of PPD-type saponins on the growth of *P. gingivalis*. **(B)** Inhibitory effect of PPT-type saponins on the growth of *P. gingivalis*. **(C)** Inhibitory effect of PPD-type saponins on the growth of *F. nucleatum*. **(D)** Inhibitory effect of PPT-type saponins on the growth of *F. nucleatum*. *p < 0.05 versus the normal control.

Because total ginsenosides mostly contain PPD- and PPT-type saponins, we assessed their antibacterial structure–activity relationships. As shown in Figure 4, both types of saponins significantly inhibited the growth of *P. gingivalis* and *F. nucleatum*, but their inhibitory effects varying.

At 0.125 mg/mL, PPD-type saponin had an inhibitory effect on *P. gingivalis* (Figure 4A), whereas PPT-type saponins were effective at 0.25 mg/mL (Figure 4B). For *F. nucleatum*, PPD-type saponins were effective at 0.5 mg/mL (Figure 4C), whereas PPT-type saponins required a concentration of 1 mg/mL (Figure 4D) to show the same effect.

Overall, the antibacterial effect of PPD-type saponins was more significant than that of PPT-type saponins, with effects on *P. gingivalis* being better.

### 3.5 Inhibitory effects of monomer saponins on *Porphyromonas gingivalis* and *Fusobacterium nucleatum*

**Figure 5:**
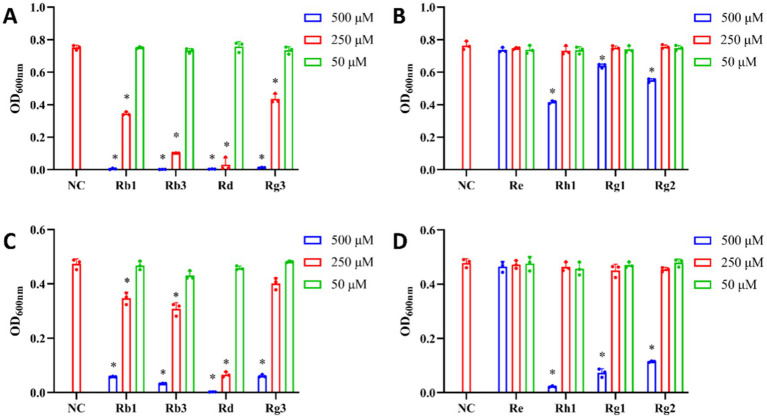
Effects of monomer saponins on the growth of *P. gingivalis* and *F. nucleatum*. **(A)** Inhibitory effect of PPD-type monomer saponins on the growth of *P. gingivalis*. **(B)** Inhibitory effect of PPT-type monomer saponins on the growth of *P. gingivalis*. **(C)** Inhibitory effect of PPD-type saponins monomer on the growth of *F. nucleatum*. **(D)** Inhibitory effect of PPT-type monomer saponins on the growth of *F. nucleatum*. *p < 0.05 versus the normal control.

To further analyze antibacterial structure–activity relationships of ginsenosides, we selected four PPD-type (Rb1, Rb3, Rd and Rg3) and four PPT-type (Re, Rh1, Rg1 and Rg2) monomer saponins to investigate. As shown in Figure 5, PPD-type monomer saponins elicited better antibacterial effects on *P. gingivalis* and *F. nucleatum* than PPT-type monomer saponins. We also found that PPD-type monomer saponins had a better inhibitory effect on *P. gingivalis* than on *F. nucleatum*. For PPD-type monomer saponins, Rb1, Rb3, Rd and Rg3 inhibited the growth of *P. gingivalis* and *F. nucleatum* in a dose-dependent manner. When the concentration reached 500 μM, the growth of *P. gingivalis* and *F. nucleatum* was almost completely inhibited. Among them, Rd had the greatest inhibitory effect on both bacteria, an effect that was clearly obvious at 250 μM. Zhou et al. also found that 400 μM Rd completely inhibited the growth of *P. gingivalis* (Zhou et al., 2022). Therefore, Rd can be considered as an ideal candidate for the treatment of periodontitis. With Rh1, Rg1 and Rg2, addition of 50 μM and 250 μM had little effect on *P. gingivalis* and *F. nucleatum*, but at 500 μM, they did inhibit the growth of both stains, especially *F. nucleatum*. However, Re had no inhibitory effect on *P. gingivalis* and *F. nucleatum*.

Based on these results, the antibacterial effect of PPD-type saponin monomer was better than that of PPT saponin monomer. Among the PPD-type monomer saponins, Rd had the best antibacterial activity. PPD- and PPT-type saponins belong to the class of tetracyclic triterpenoid saponins, with their sugar moieties being correlated with the biological activities of ginsenosides. The main difference between PPD- and PPT-type monomeric saponins is their terminal glycosyl groups at C-3, C-6, and C-20. The glycosyl groups of PPD-type saponins Rb1, Rb3, Rd and Rg3 are located at C-3 and C-20 positions, whereas glycosyl groups on PPT-type saponins Re, Rh1, Rg1 and Rg2 are located at their C-6 and C-20 positions. The C-3 terminal glycosyl groups of PPD-type saponins Rb1, Rb3, Rd and Rg3 are all Glc (*β*-1,2) Glc-. The sugar group at C-20 of Rb1 is Glc (*β*-1,6) Glc-, Rb3 is Xyl (*β*-1,6) Glc-, Rd is Glc- and Rg3 does not contain any sugar groups. This difference in the C-20 glycosylation may be one reason why the antibacterial activity of Rd is better than that of the other monomer saponins. The PPT-type monomer saponin Rh1 has only one Glc- at the C-6 position, Rg1 has one Glc- at the C-6 and one Glc- at the C-20 position, Rg2 has Rha (*α*-1,2) Glc- at the C-6 position, and Re has Rha (*α*-1,2) Glc- at the C-6 and Glc- at the C-20 position, respectively. The presence of Rha (*α*-1,2) Glc- may affect the antibacterial activity of ginsenosides. In addition, the difference in the position of sugar chains may also explain differences in the antibacterial activities of ginsenosides.

Some studies had found that the physiological activity of ginsenosides may be related to their structure. The anti-cancer activity is negatively correlated with the number of sugar residues. Ginsenosides with more sugar residues had no significant anti-cancer activity, which may be due to the polar hydroxyl group in the sugar part. It reduced the hydrophobicity of ginsenosides and made them difficult to cross the cell membrane (Elsaman et al., 2024). In this study, the number of sugar groups may also affect the antibacterial activity of ginsenosides. Compared with ginsenoside Rb1, ginsenoside Rd has only one Glc- at the C-20 position, but it exhibits better antibacterial activity than Rb1.

### 3.6 Effects of total ginsenosides on biofilm formation

**Figure 6:**
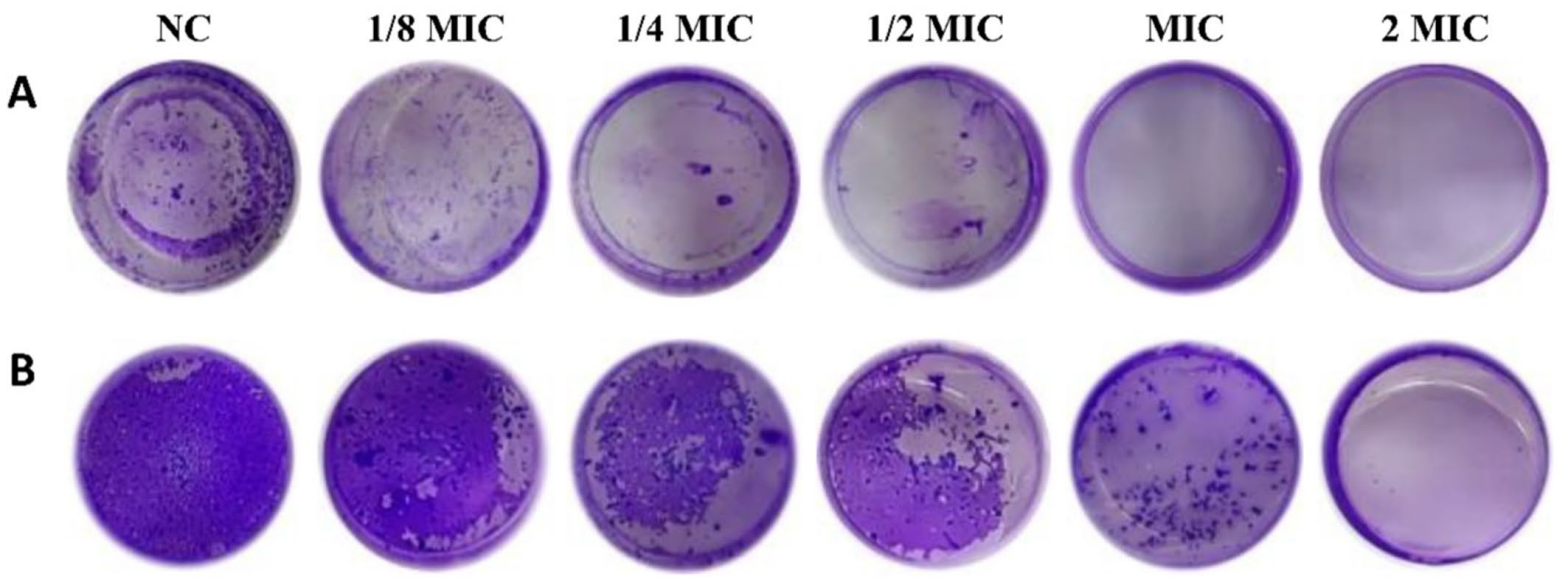
Crystal violet staining. **(A)** Biofilm formation after treatment of *P. gingivalis* with different concentrations of total ginsenosides. **(B)** Biofilm formation after treatment of *F. nucleatum* with different concentrations of total ginsenosides.

The biofilm of pathogenic bacteria is the initiating factor of periodontal disease. Biofilm formation can lead to chronic infection that is unaffected by host immunity and resistant to antimicrobials (Nishikawa et al., 2024). Therefore, we studied the effects of ginsenoside on biofilm formation by pathogenic bacteria. For this, *P. gingivalis* and *F. nucleatum* were cultured under anaerobic conditions for 3 days to form biofilms. At the same time, different concentrations of ginsenosides were added to *P. gingivalis* and *F. nucleatum* and cultured under the same conditions. After crystal violet staining, biofilm formation was observed and quantified. As seen in Figure 6, biofilm formation decreased gradually with increasing ginsenoside concentration (Flemming et al., 2016). Biofilm formation with *F. nucleatum* was greater than that with *P. gingivalis. F. nucleatum* is the most abundant bacterium in the human gingival sulcus and can co-aggregate with other bacteria, allowing it to play a central role in biofilm formation. When the concentration of total ginsenosides was at the MIC, *P. gingivalis* did not produce biofilm (Figure 6A), and when the concentration of total ginsenosides was 2 MIC, *F. nucleatum* also did not produce biofilm (Figure 6B). These results demonstrate that total ginsenosides can inhibit the growth of *P. gingivalis* and *F. nucleatum* by reducing biofilm formation.

Total ginsenosides may inhibit biofilm growth by interfering with quorum sensing (QS) among bacteria. QS system significantly enhanced the pathogenicity of periodontal pathogens by regulating virulence factor secretion, biofilm formation and immune escape (Bridges and Bassler, 2019). By sensing self-inducers, bacteria can judge the density of bacteria and changes in the surrounding environment, and can activate the regulatory expression of a series of genes to regulate the population behavior of bacteria. When ginsenosides acted on periodontal pathogens, the expression of virulence regulation genes was down-regulated, which interfered with QS process among bacteria. Previous report found that the expression of virulence genes (*fimA* and *kgp*) was significantly reduced after treating *P. gingivalis* with Rd (Zhou et al., 2022).

### 3.7 Effect of total ginsenosides on CSH of *Porphyromonas gingivalis* and *Fusobacterium nucleatum*

**Figure 7:**
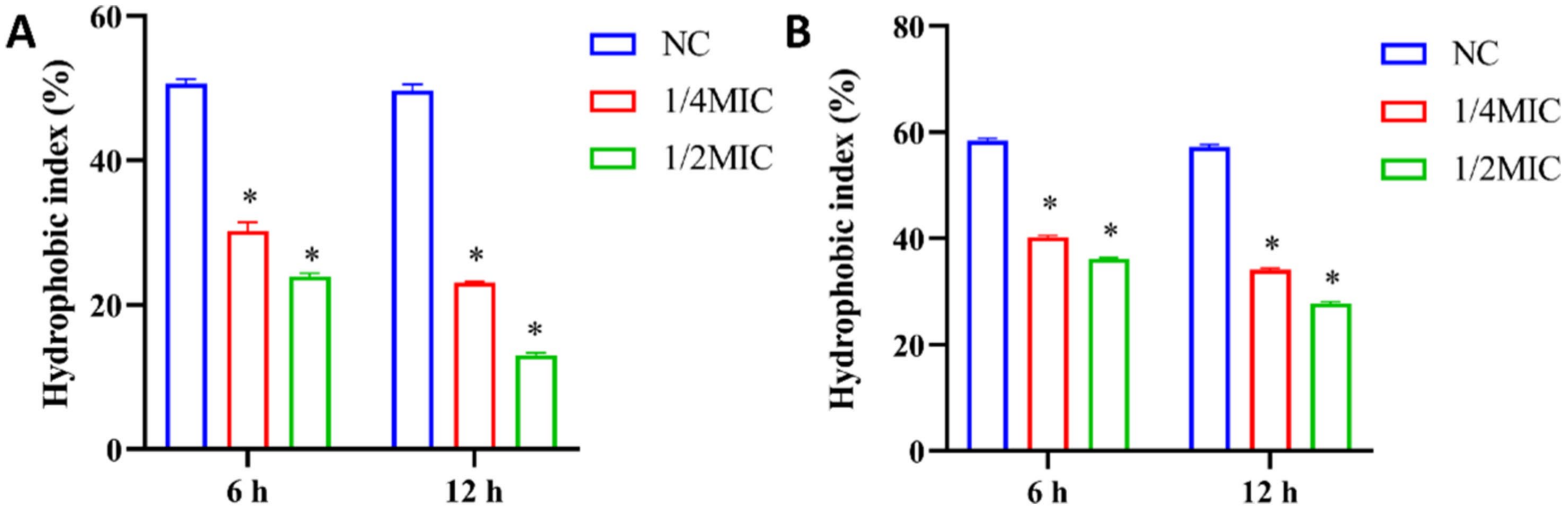
Analysis of surface hydrophobicity after treatment of *P. gingivalis* and *F. nucleatum* with total ginsenoside. **(A)** The surface hydrophobicity of *P. gingivalis*. **(B)** The surface hydrophobicity of *F. nucleatum*. *p < 0.05 versus the normal control.

Hydrophobic interaction is a factor that affects bacteria-host reactions and adhesion that is a crucial step in formation of dental plaque biofilm that impacts the pathogenicity of dental plaque (Ellepola and Samaranayake, 1998). The decrease of bacterial surface hydrophobicity can lead to the decrease of adhesion. To further explore how ginsenosides destroy biofilms, the surface hydrophobicity of bacteria was determined. In our study, the surface hydrophobicity of *P. gingivalis* and *F. nucleatum* treated with different concentrations of total ginsenosides was determined by MATH method (Lee and Lee, 2019). The hydrophobic index of the two strains was significantly reduced upon treatment with 1/4 MIC and 1/2 MIC of total ginsenosides and was gradually decreased with increasing ginsenoside concentration (Figure 7). Our results indicated that ginsenosides can significantly inhibit surface hydrophobicity of *P. gingivalis* and *F. nucleatum*, which, in turn, reduced bacterial adhesion and thus reduced bacterial ability to form biofilms.

Hydrophobic bacteria are more likely to adsorb onto hydrophobic surfaces through hydrophobic-hydrophobic interactions, thereby initiating biofilm formation (Pagedar et al., 2010; Di Ciccio et al., 2015). CSH plays an important role in biofilm formation and intercellular adhesion, and is the core of biofilm formation and development (Vandana and Das, 2023). Studies had shown that some natural drugs can affect the formation of biofilms by destroying cell hydrophobicity, thereby reducing the pathogenicity of pathogens. For example, apigenin-7-O-glucoside exerted strong anti-biofilm activity by inhibiting CSH (Pei et al., 2023).

### 3.8 Effects of total ginsenosides on EPS content in *Porphyromonas gingivalis* and *Fusobacterium nucleatum*

**Figure 8:**
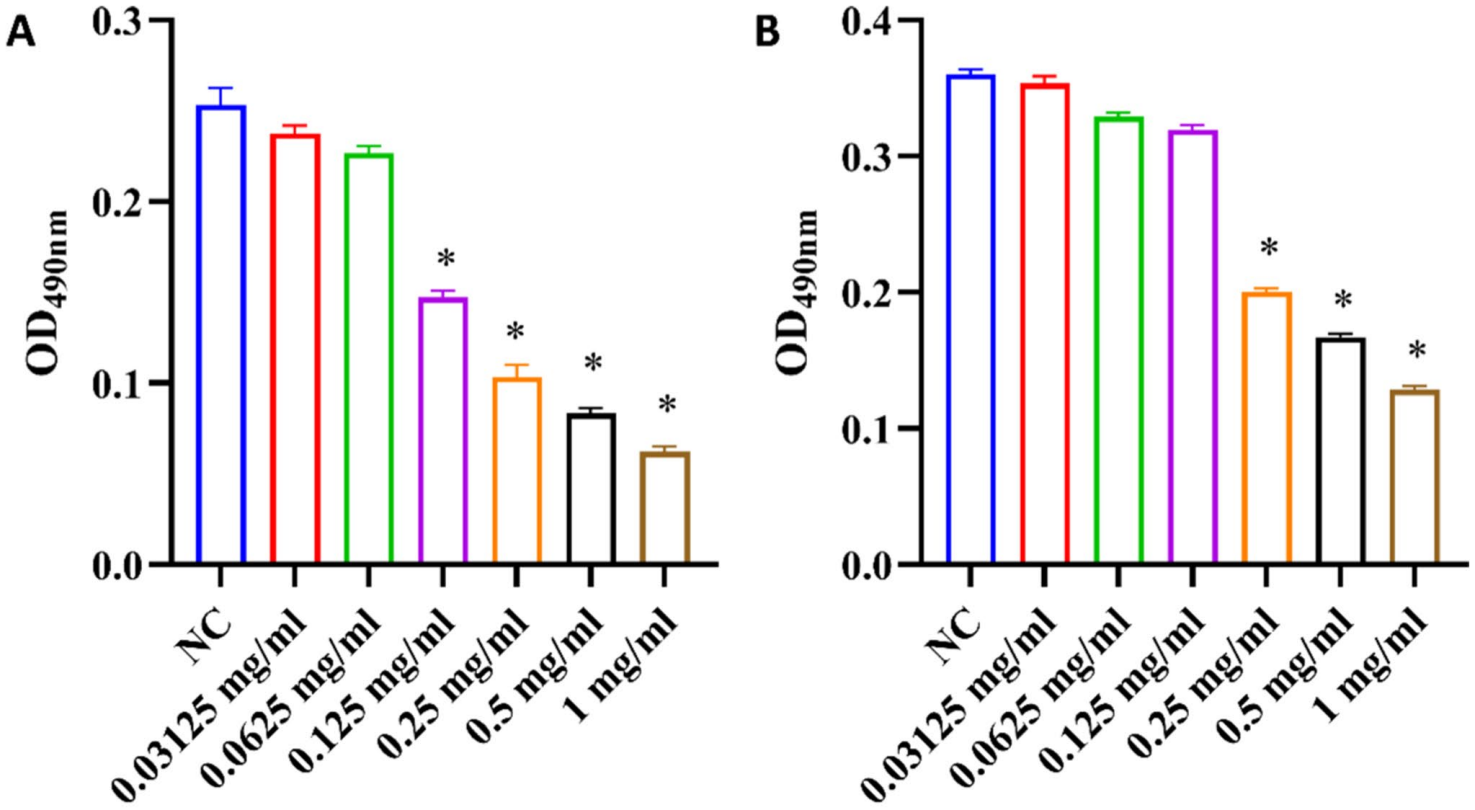
Changes of EPS content in biofilms upon treatment of *P. gingivalis* and *F. nucleatum* with total ginsenoside. **(A)** Changes of EPS content of *P. gingivalis*. **(B)** Changes of EPS content of *F. nucleatum*. *p < 0.05 versus the normal control.

The EPS secreted by bacteria may contain hydrophobic components such as lipids, hydrophobic proteins, which cooperate with the hydrophobicity of the bacterial surface to enhance the stability of the biofilm structure (Ma et al., 2022). EPS promotes the adhesion and aggregation of bacteria on the tooth surface and can protect bacteria from the influence of the external environment (Li et al., 2023). This provides energy and carbon sources for microorganisms and is crucial for pathogenic bacteria (Felz et al., 2019). In this study, the content of EPS in biofilms was determined by using the phenol-sulfuric acid method. As shown in Figure 8, the content of EPS decreased with increasing ginsenoside concentration. Production of EPS in *P. gingivalis* and *F. nucleatum* biofilms was significantly inhibited by total ginsenoside at 0.125 mg/mL and 0.25 mg/mL, respectively. Our study found that total ginsenosides reduce the amount of EPS in biofilms, and thereby reduced biofilm formation by *P. gingivalis* and *F. nucleatum*, accounting for the observed antibacterial effects.

Bacteria with biofilms are highly resistant to antibiotics and host immune defense mechanisms. As a chronic disease, the development of periodontitis was related to the accumulation of biofilm of periodontal pathogens (Kwon et al., 2021). In this study, ginsenosides showed a significant inhibitory effect on the growth of *P. gingivalis* and *F. nucleatum*. Further studies had found that ginsenosides can destroy the formation of biofilms by reducing cell surface hydrophobicity and reducing EPS secretion, reducing adhesion between pathogens, and reducing their pathogenicity. As a potential periodontitis treatment drug, ginsenosides can be used in the future to develop gels, mouthwashes or sustained-release materials that directly act on periodontal pockets to enhance efficacy, or in combination with traditional antibiotics to achieve better therapeutic effects. In the future, Nano-delivery technology can be combined to optimize the bioavailability and targeting of ginsenosides, providing a new strategy for the treatment of periodontitis.

## 4 Conclusion

In conclusion, total ginsenosides have significant inhibitory effects on the growth of *P. gingivalis* and *F. nucleatum*. For *P. gingivalis*, the MIC was 1 mg/mL, and the MBC was 1 mg/mL. For *F. nucleatum*, the MIC and MBC were 1 mg/mL and 2 mg/mL, respectively. The antibacterial effect of PPD-type saponins was more significant than that of PPT-type saponins, and the monomer saponin Rd in the PPD-type had the best antibacterial effect. Rd at 250 μM inhibited the growth of periodontal pathogens. Mechanistic analyses showed that total ginsenosides reduced the surface hydrophobicity and EPS biofilm content of periodontal pathogens, thereby reducing the adhesion of periodontal pathogens and effectively inhibiting formation of biofilms of *P. gingivalis* and *F. nucleatum* pathogens.

## Data Availability

The original contributions presented in the study are included in the article/supplementary material, further inquiries can be directed to the corresponding authors.
